# Pharmacological or genetic orexin1 receptor inhibition attenuates MK-801 induced glutamate release in mouse cortex

**DOI:** 10.3389/fnins.2014.00107

**Published:** 2014-05-20

**Authors:** Leah Aluisio, Ian Fraser, Tamara Berdyyeva, Volha Tryputsen, Brock T. Shireman, James Shoblock, Timothy Lovenberg, Christine Dugovic, Pascal Bonaventure

**Affiliations:** Janssen Pharmaceutical Research and Development, LLCSan Diego, USA

**Keywords:** orexin, orexin 1 receptor, glutamate, MK-801, cortex, biosensor

## Abstract

The orexin/hypocretin neuropeptides are produced by a cluster of neurons within the lateral posterior hypothalamus and participate in neuronal regulation by activating their receptors (OX1 and OX2 receptors). The orexin system projects widely through the brain and functions as an interface between multiple regulatory systems including wakefulness, energy balance, stress, reward, and emotion. Recent studies have demonstrated that orexins and glutamate interact at the synaptic level and that orexins facilitate glutamate actions. We tested the hypothesis that orexins modulate glutamate signaling via OX1 receptors by monitoring levels of glutamate in frontal cortex of freely moving mice using enzyme coated biosensors under inhibited OX1 receptor conditions. MK-801, an NMDA receptor antagonist, was administered subcutaneously (0.178 mg/kg) to indirectly disinhibit pyramidal neurons and therefore increase cortical glutamate release. In wild-type mice, pretreatment with the OX1 receptor antagonist GSK-1059865 (10 mg/kg S.C.) which had no effect by itself, significantly attenuated the cortical glutamate release elicited by MK-801. OX1 receptor knockout mice had a blunted glutamate release response to MK-801 and exhibited about half of the glutamate release observed in wild-type mice in agreement with the data obtained with transient blockade of OX1 receptors. These results indicate that pharmacological (transient) or genetic (permanent) inhibition of the OX1 receptor similarly interfere with glutamatergic function in the cortex. Selectively targeting the OX1 receptor with an antagonist may normalize hyperglutamatergic states and thus may represent a novel therapeutic strategy for the treatment of various psychiatric disorders associated with hyperactive states.

## Introduction

Orexins, also known as hypocretins, are two peptides (orexin A and B) derived from a single precursor produced exclusively in the hypothalamus (de Lecea et al., 1998). Orexins participate in neuronal regulation by activating their receptors (OX1 and OX2 receptors). The orexin system is emerging as a powerful integrator of multiple physiological functions including sleep/wakefulness states, energy balance, stress, reward, and emotion (for latest review see Li et al., 2014). Orexin neurons project broadly throughout the whole brain and can be modulated by multiple humoral signals and neuronal inputs. Several key areas from the limbic system (including the bed of the stria terminalis, the amygdala, and the medial septum) send axons to orexin cells (Sakurai et al., 2005). The majority of synapses on orexin somata and dendrites are asymmetric with small, round, clear vesicles that reflect excitatory neural transmission (Zhang et al., 2005). Orexins and glutamate interact at the synaptic level where orexins facilitate glutamatergic actions. In turn, glutamatergic neurons regulate orexinergic neuronal activity via presynaptic facilitation of glutamate release (Li et al., 2002). Early *in vitro* experimental data have shown that orexin A increased glutamate release from hypothalamic slices (van den Pol et al., 1998). In rats, intravenous administration of orexin A increased glutamate release in the amygdala, a brain region known to express OX receptors, but not in the cerebellum where OX receptors are not expressed (John et al., 2003). Intravenous injection of orexin A also increased glutamate release in the locus coeruleus (Kodama and Kimura, 2002) and it has been suggested that orexin neurons and NMDA receptors interact together in the control of the locus coeruleus noradrenergic activity (Tose et al., 2009). These actions might be mediated via the OX1 receptors since this OX receptor subtype is exclusively expressed in the locus coeruleus (Trivedi et al., 1998). In the present study, we tested the hypothesis that orexin modulates glutamate signaling in cortex via OX1 receptors by monitoring levels of glutamate in the prefrontal cortex of freely moving mice. In contrast to the locus coeruleus, in cortex both OX receptor subtypes are expressed (Marcus et al., 2001) and therefore the OX1 receptor might not be the only receptor involved. We used an enzyme coated biosensors to monitor real time change in glutamate release (Uslaner et al., 2012). MK-801, an NMDA receptor antagonist, was administered to indirectly disinhibit pyramidal neurons and increase glutamate release in cortex. In the first experiment we investigated the effect of transient inhibition of the OX1 receptor by systemic administration of the selective and brain penetrant OX1 receptor antagonist GSK-1059865 (Gozzi et al., 2011) on MK-801 induced glutamate release in the cortex of wild-type mice. In a second experiment, we investigated the effect of permanent inhibition of OX1 receptors by comparing the effect of MK-801 on glutamate release in the cortex of wild-type vs. OX1 receptor knockout mice.

## Methods

All animal experimental procedures were performed in accordance with the Guide for the Care and Use of Laboratory Animals adopted by the US National Institutes of Health.

Male wild-type mice and fully backcrossed OX1 receptor knockout mice of the same age (8–12 weeks old) and strain (Jackson Labs C57/Bl6) were singly housed at the time of experimentation. Biosensor technology was used to measure real-time change of glutamate release in the cortex of freely moving mice as previously described (Uslaner et al., 2012). Each mouse was given a subcutaneous injection of Buprenex (0.1 mg/kg, buprenorphine hydrochloride; Reckitt Benckiser Pharmaceuticals Inc., Richmond, VA) 5 min prior to anesthesia. Animals were anesthetized with an isoflurane/air mixture and stereotaxically implanted with a guide cannula (BAS) in the prefrontal cortex (+1.54 mm anterior, 0.5 mm lateral, right hemisphere and 1.4 mm ventral to Bregma, Supplemental Data Figure S1). A grounding screw attached to the “headmount connector” was inserted slightly anterior to the interaural line. The guide cannula and head mount connector were secured in place with dental cement. Animals were allowed at least 5 days to recover from surgery prior to experimentation.

The glutamate sensor (model #7004 Pinnacle Technology Inc., Lawrence, KS) specification and hardware setup has been described in detail previously (Naylor et al., 2011). Briefly, glutamate biosensors act through enzyme mediated processing. Glutamate is converted to hydrogen peroxide and detected by oxidation at the Pt-Ir electrode. A selectively passive membrane allows for exclusion of electroactive interferents. Prior to sensor insertion, each biosensor was calibrated *in vitro* to verify glutamate sensitivity and interference rejection. The biosensors extended beyond the guide cannulas by 1 mm. The afternoon prior to experimentation, under light isoflurane anesthesia, biosensors were inserted into the guide cannula of each animal which was then returned to its home cage for sensor equilibration. Data acquisition started immediately and the animals were maintained connected to the acquisition hardware overnight. The sensor signal was processed by the 8401 Data Acquisition System and data acquisition, storage, and analysis were performed by the Pinnacle Acquisition Laboratory software suite. Experimentation was conducted the following day between 8:00 am and 3:00 pm (light cycle) in a controlled environment.

A group of wild-type mice was administered with saline (S.C.) and monitored for a 90- min baseline period. Each animal was then pretreated with the selective OX1 receptor antagonist (GSK-1059865, 10 mg/kg S.C.) or vehicle 30 min prior to the injection of MK-801 (0.178 mg/kg, S.C.). The effect of GSK-1059865 on its own (in the absence of MK-801) was also assessed in an across study subject design. At the end of each test session, naïve animals to MK-801 received MK-801 (0.178 mg/kg S.C.) as a positive control test.

GSK-1059865 was synthetized at Janssen Research and Development LLC and was formulated in 30% sulfobutylether(7)-β-cyclodextrin (SBE) or 5% Pharmasolve, 20% Cremaphor and 75% D5W. MK-801 was purchased from Sigma and formulated in saline. All injection volumes were 10 ml/kg. The doses of GSK-1059865 (10 mg/kg) and MK-801 (0.178 mg/kg) were selected based on literature data where biological activity has been reported (Bonaventure et al., 2011; Gozzi et al., 2011; Uslaner et al., 2012).

The study was done in the set of three experiments. For each animal the area under the curve of the baseline change in glutamate release over 1 h post MK-801 stimulation was calculated. To assess whether the mean area under the curve differs for the two treatment groups (GSK-1059865 and vehicle treated mice) we used One-Way ANOVA, blocking for the experiment effect. Two-sided paired *t*-test was used to assess the effect of GSK-1059865 (vs. vehicle) on the area under the curve of glutamate release over 1 h time period.

A second experiment using OX1 receptor knockout and wild-type mice was conducted as a within subject study design. All animals received a subcutaneous saline injection, and were treated with MK-801 (0.178 mg/kg S.C.) 90 min later. The area under the curve was calculated for each animal and the two-sample one-sided *t*-test was performed to compare the area under the curve of changes in glutamate from baseline over 1 h between MK-801 stimulated OX1 receptor knockout and wild-type mice.

Changes in glutamate concentration were recorded as current (nA) every second for at least 60 min following the last drug administration. The data were averaged into 2-min bins and glutamate concentrations were calculated using post calibration values.

At the completion of the experiment, each sensor was removed from the guide cannula and calibrated *in vitro* for glutamate sensitivity and interference rejection of ascorbate at 37°C in a circulating water bath. At the end of the experimentation, the brains were coronally sectioned and sensor location was visually verified. Sensor placements which were outside the targeted area by ±0.2 mm were not included.

## Results

**Figure 1:**
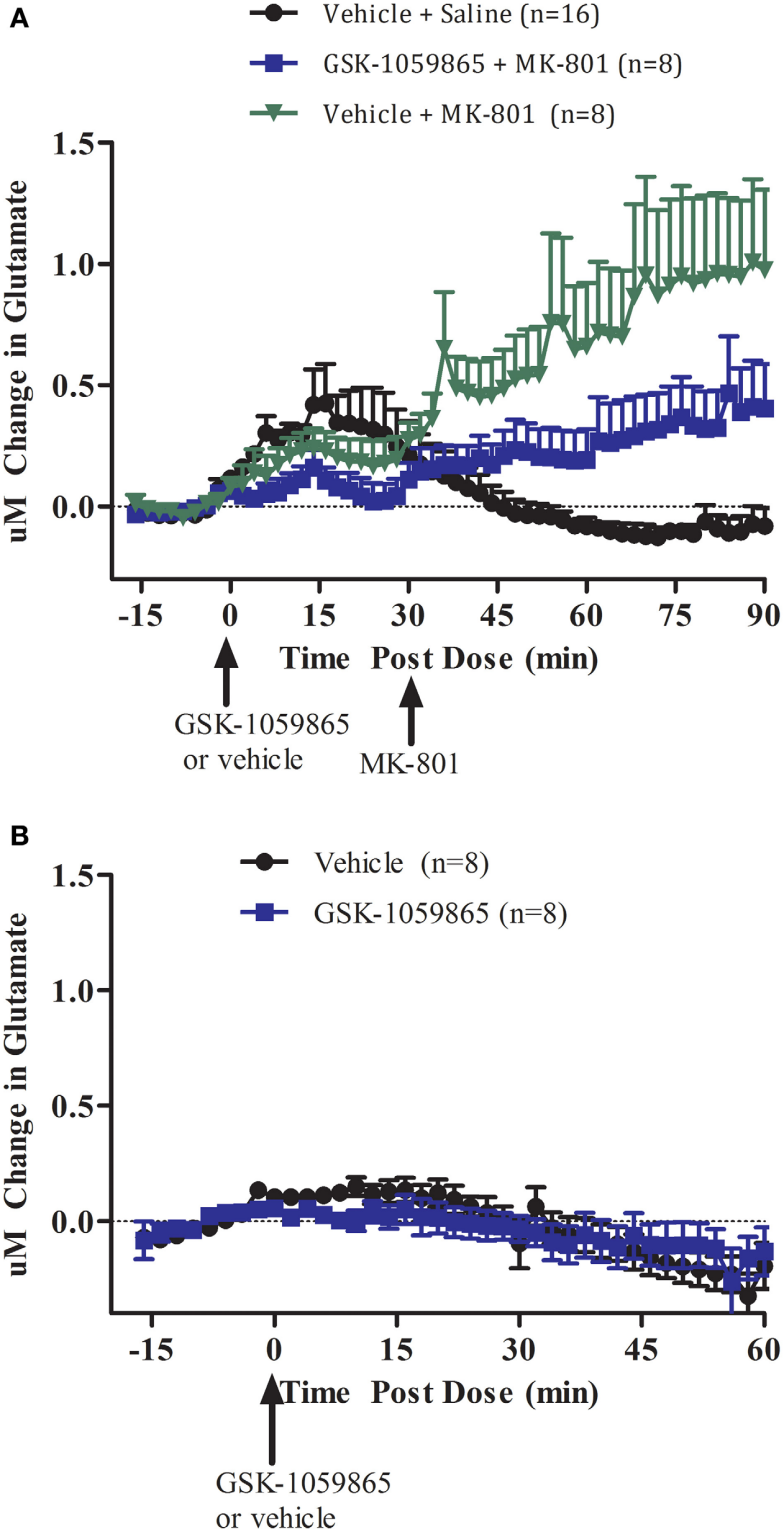
**(A)** Effect of GSK-1059865 (10 mg/kg S.C.) on MK-801 (0.178 mg/kg, S.C.) induced glutamate release in the cortex of freely moving wild-type mice (within subject study design). GSK-1059865 was administered at *t* = 0 and MK-801 was administered at *t* = 30 min. **(B)** Effect of GSK-1059865 (10 mg/kg S.C.) on glutamate release in the cortex of freely moving wild-type mice (across subject study design). GSK-1059865 was administered at *t* = 0. Real-time measurements of glutamate were conducted before and after compound administration. Results are expressed as change in glutamate concentrations as mean ± s.e.m. GSK-1059865 did not affect glutamate levels *per se* (*p* = 0.118) but attenuated MK-801-induced increase in glutamate release (*p* = 0.037).

We first investigated the effect of transient inhibition of the OX1 receptor with a selective brain penetrant OX1 receptor antagonist on MK-801 induced glutamate release in the cortex of wild-type animal (Figure 1A). In wild-type mice, GSK-1059865 (10 mg/kg, S.C.) significantly attenuated cortical glutamate release elicited by MK-801 (0.178 mg/kg S.C.) (Figure 1A, treatment group: *F* = 5.537, *df* = 1, *p* = 0.037; block: *F* = 3.677, *df* = 2, *p* = 0.057). As compared to vehicle GSK-1059865 did not affect glutamate release *per se* (Figure 1B, *t* = 1.784, *df* = 7, *p* = 0.118).

**Figure 2:**
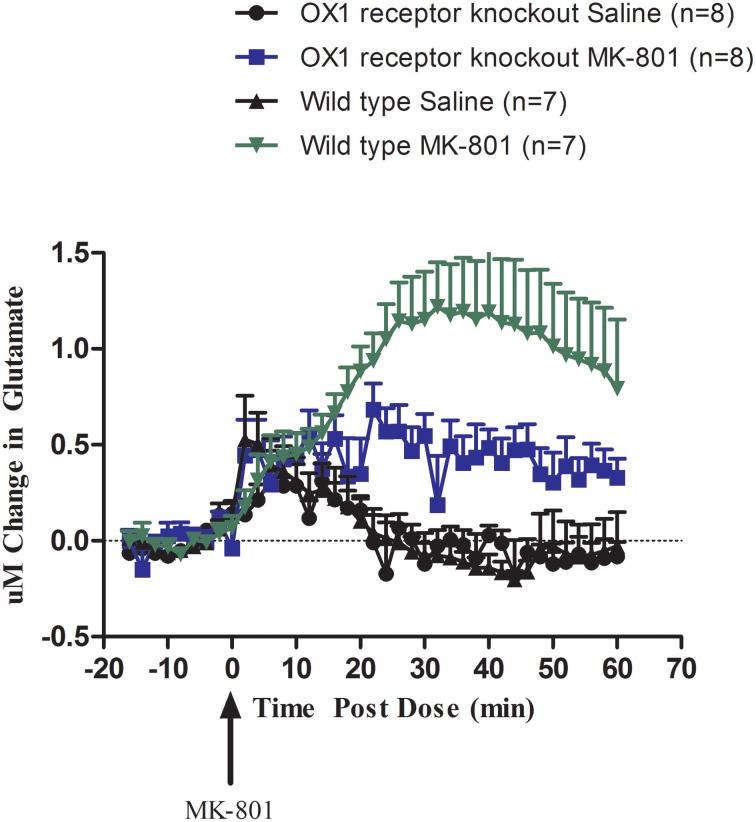
**Effect of MK-801 (0.178 mg/kg, S.C.) on glutamate release in the cortex of freely moving OX1 receptor knockout mice and wild-type mice (within subject study design)**. Real-time measurements of glutamate were conducted before and after compound administration (MK-801 was administered at *t* = 0). Results are expressed as change in glutamate concentrations as mean ± s.e.m. MK-801 response in OX1 receptor knockout mice was attenuated compared to wild-type mice (*p* = 0.042).

We then investigated the effect of permanent inhibition of the OX1 receptor by comparing the effect of systemic administration of MK-801 (0.178 mg/kg S.C.) on glutamate release in the cortex of wild-type and OX1 receptor knockout mice (Figure 2). Compared to wild-type mice, OX1 receptor knockout mice had a blunted glutamate release response to MK-801 (*t* = 1.981, *df* = 9, *p* = 0.04). MK-801 still induced an increase in glutamate release in OX1 receptor knockout mice but the magnitude of this increase was in a range of about 30–50% of the release observed in wild-type animals after 20 min post treatment.

## Discussion

In this study we used a pharmacological and genetic approach to investigate the functional interaction between the OX1 and NMDA receptors using MK-801 induced glutamate release in mouse frontal cortex. In wild-type mice, transient inhibition of the OX1 receptor with a selective OX1 receptor antagonist significantly attenuated excessive cortical glutamate release elicited by MK-801. Systemic administration of the selective OX1 receptor antagonist had no effect on glutamate release on its own. Permanent inhibition of OX1 receptor in OX1 receptor knockout mice resulted in a diminished glutamate release evoked by MK-801 comparable to the response obtained with the OX1 receptor antagonist. Therefore, these data ruled out the possibility of a direct pharmacokinetic interaction between MK-801 and GSK-1059865 in wild-type mice.

Brain penetration and biological activity of GSK-1059865 has been recently demonstrated (Gozzi et al., 2011) and later confirmed in our lab in a similar dose range (Dugovic et al., 2014).

The biosensor technology used to monitor glutamate release in this study is well established. Noteworthy, the effects of vehicle on glutamate release are substantially greater in the first experiment (Figure 1A) when compared to the second experiment (Figure 1B). Inter-animal variability is common during animal handling and injections as glutamate release has been linked to an animal's arousal state and individual stress response (Westerink and Cremers, 2007). Previous studies have shown that MK-801 increased glutamate release in the rat cortex (Lopez-Gil et al., 2007; Bonaventure et al., 2011) and nucleus accumbens (Uslaner et al., 2012). The increase in cortical glutamatergic transmission elicited through blockade of an excitatory glutamate receptor (NMDA) results from an indirect effect of the NMDA receptor antagonist on GABAergic interneurons (Krystal et al., 2003). NMDA receptor antagonists attenuate the tonic activation of inhibitory neurons (GABA) most likely in the hippocampus and/or thalamus resulting in a disinhibition of glutamatergic input in the medial prefrontal cortex. Noteworthy, MK-801 also acts as a nicotinic acetylcholine receptor antagonist in addition to its NMDA receptor antagonistic properties (Amador and Dani, 1991). Ketamine, another NMDA receptor antagonist used in the clinic, has been reported to increase glutamate release independently from its effect on locomotor activity in mice (Schobel et al., 2013). Both MK-801 and ketamine have been used as animal models for psychosis at doses that produce substantial increase of glutamate release (Large, 2007). Interestingly, ketamine has been reported to have rapid antidepressant properties at low dosage in patients resistant to antidepressive treatment (Salvadore and Singh, 2013). The mechanism for this antidepressant action is not fully elucidated, in particular in reference to its effect on glutamate release.

In this study, we conducted the experiments during the light cycle when orexin neurons are less active (Lee et al., 2005). Interestingly, the OX1 receptor antagonist only partially blunted the effect of MK-801 on glutamate release. During the dark phase when orexin neurons are more active we postulate that the effect of the OX1 receptor antagonist on blunting MK-801 induced glutamate release might be more pronounced. However, other neurochemicals and/or receptors might be involved in this complex phenomenon as suggested by the data obtained in OX1 receptor knockout mice where MK-801 response was only partially diminished compared to the response obtained in wild-type animals. The partial response observed in knockout mice or after antagonist administration supports the view that the action of orexin is modulatory.

Evidences for glutamate and orexins co-localization and co-release from orexin terminals in the locus coeruleus have been presented (Henny et al., 2010). Orexins neurons originating from the hypothalamus and projecting to the cortex contain both orexins and glutamate. Interactions between orexinergic and glutamatergic neurons have been described in the locus coeruleus, amygdala and in the bed nucleus of the stria terminalis (John et al., 2003; Tose et al., 2009; Lungwitz et al., 2012). In locus coeruleus, the OX1 receptor is the only OX receptor present (Marcus et al., 2001). In amygdala, bed nucleus of the stria terminalis, and cortex both OX receptors are present (Trivedi et al., 1998; Marcus et al., 2001). Future studies should examine the potential contribution of the OX2 receptor in addition to the role of the OX1 receptor.

Activity of orexin neurons is increased under stressful stimuli leading to release of more orexins in terminal fields located in the limbic system. It has been postulated that an abnormal persistence of excessive release of orexins could lead to pathological anxiety or panic attacks (Johnson et al., 2012). Emerging data indicate that the anxiogenic properties of orexins are mediated through interaction with the glutamatergic system and that engagement of the OX1 receptor is needed (Lungwitz et al., 2012). The present study focused on the orexins/glutamate interaction in cortex, and this important interaction is likely to be involved with arousal during stress or attention to a stressor. Contribution of the OX2 receptor for the orexin/glutamate interaction still needs to be studied but from a therapeutic standpoint it is well established that blocking the OX2 receptor will lead to an hypnotic effect (Hoyer and Jacobson, 2013; Dugovic et al., 2014) whereas in contrast selective OX1 receptor blockade does not alter spontaneous sleep. Selective targeting of the OX1 receptor with an antagonist may normalize hyperglutamatergic states without hypnotic effect and thus may represent a novel therapeutic strategy for the treatment of various psychiatric disorders associated with hyperactive states.

## Author contributions

Pascal Bonaventure designed research, analyzed data, and wrote manuscript. Leah Aluisio analyzed data and edited manuscript. Ian Fraser and Tamara Berdyyeva conducted research and analyzed results. Christine Dugovic and James Shoblock participated in research design and edited manuscript. Volha Tryputsen performed the statistical analysis. Brock T. Shireman provided compounds. Timothy Lovenberg participated in research design.

### Conflict of interest statement

All the authors are full-time employee of Janssen Research and Development, L.L.C.
