# Supplementary material for: Pharmacological or genetic orexin1 receptor inhibition attenuates MK-801 induced glutamate release in mouse cortex
Source: Front Neurosci. 2014 May 20;8:107. doi: 10.3389/fnins.2014.00107 (PMC4033200; doi:10.3389/fnins.2014.00107)
Supplement: Supplementary file 1 [file DataSheet1.PDF]

Supplemental Data Figure 1

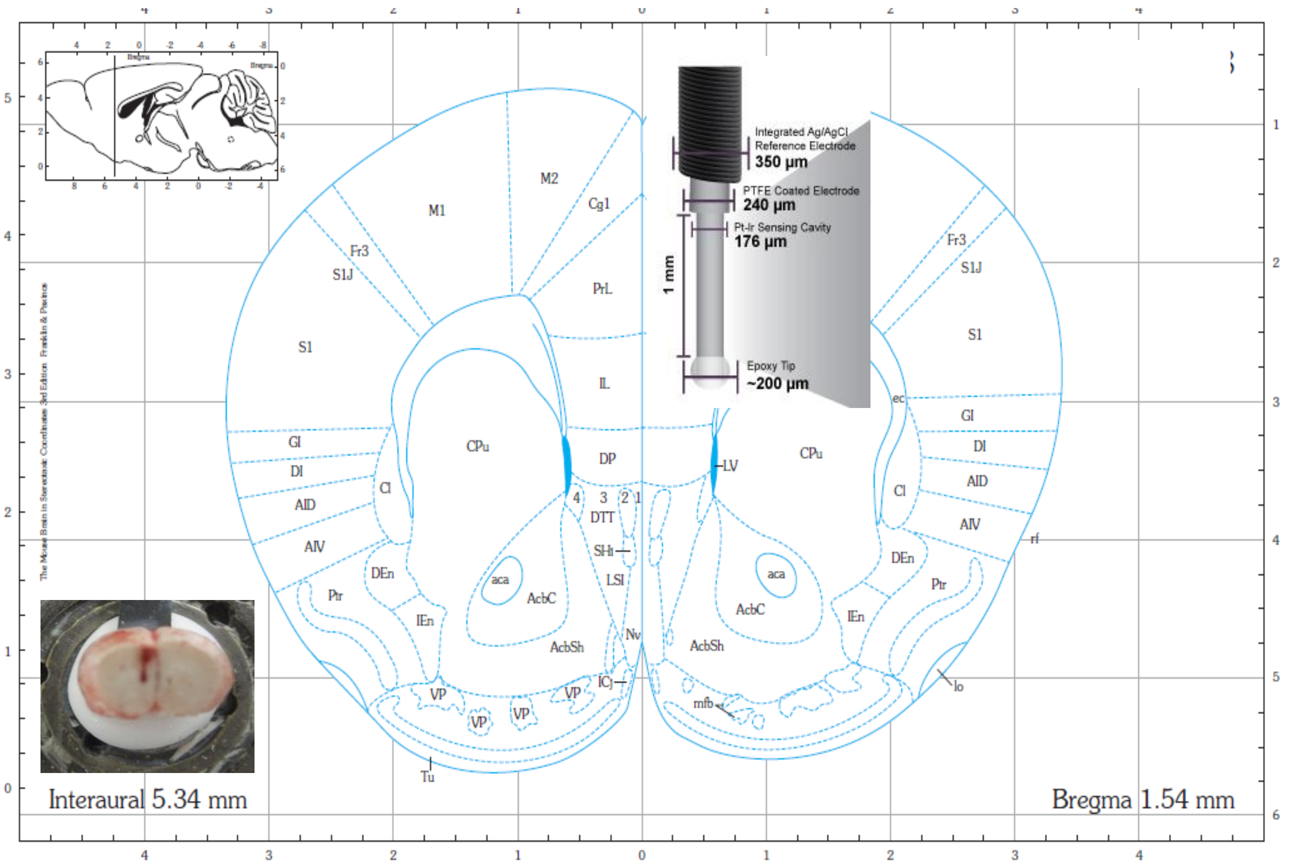

Placement of the guide cannula in the prefrontal cortex. Adapted from The Mouse Brain in Stereotaxic Coordinates K.B.J. Franklin and G. Paxinos, 3rd Edition, 2008, Academic Press
